# Changes in Risk Factors and Exercise Capacity After Cardiac Rehabilitation and Its Effect on Hospital Readmission

**DOI:** 10.5812/ircmj.4899

**Published:** 2014-05-05

**Authors:** Farid Najafi, Mehdi Nalini, Mohammad Reza Nikbakht

**Affiliations:** 1 Research Center for Environmental Determinants of Health, Kermanshah University of Medical Sciences, Kermanshah, IR Iran; 2 Imam Ali Heart Center, Kermanshah University of Medical Sciences, Kermanshah, IR Iran; 3 School of Pharmacy, Kermanshah University of Medical Sciences, Kermanshah, IR Iran

**Keywords:** Rehabilitation, Exercise Tolerance, Patient Readmission, Risk Factors

## Abstract

**Background:**

Despite the positive outcomes reported with cardiac rehabilitation (CR), its impacts have been reported to be different from a region or country to another, which may be due to the different contents of rehabilitation programs.

**Objectives:**

To investigate the effect of CR on cardiovascular risk factors.

**Patients and Methods:**

This is a retrospective cohort study on the data from Imam Ali Cardiac Rehabilitation Center in Kermanshah province, Iran from 2001 to 2008. We used paired t-test to evaluate the effect of CR on cardiovascular risk factors. Logistic regression or t-test (unequal variance) were used to assess the factors influencing re-admission (due to cardiac problems). The relationship between different variables and death was studied using univariate cox proportional hazard. P values < 0.05 were considered significant for all analyses.

**Results:**

Out of 504 patients who completed rehabilitation, a total of 499 were analyzed. These 499 patients consisted of 383 men and 116 women. All anthropometric measurements, blood lipids (except HDL cholesterol), systolic and diastolic blood pressure, depression, anxiety and exercise capacity improved after rehabilitation (P < 0.05 for all cases). The improvement was observed in both sexes. A total of 39 patients were re-admitted to hospital after rehabilitation. Being female (OR = 2.40; 95%CI: 1.22-4.68) and history of diabetes (OR = 2.04; 95%CI: 1.04-4.02) increased the risk of re-admission significantly. Patients who were readmitted had higher anthropometric measurements at the beginning and the end of the program. Moreover, the initial exercise capacity of readmitted patients was lower than those who were not readmitted. After a maximal follow-up period of 6.3 years (median = 2.99 years), only eight patients expired (survival rate: 97.5%; 95%CI: 94.7-98.8). None of the variables in our study was significantly related to the survival rate.

**Conclusions:**

The comprehensive CR program in Imam Ali Center efficiently reduces cardiovascular risk factors and improves exercise capacity.

## 1. Background

Cardiovascular diseases constitute the number one cause of death worldwide. Cerebral and cardiac ischemic diseases are the two most common causes of death in all countries (whether high- or low-income) and thus it has been estimated that they will continue to top the list of mortality causes until 2030 (1). In Iran and other developing countries which are experiencing an epidemiologic transition, with the disease pattern shifting from communicable to non-communicable and chronic diseases, the prevalence of cardiac disease is rising rapidly. According to the first study on burden of diseases in Iran in 2003, cardiovascular diseases constitute the third cause of disability adjusted life years (DALYs) after intentional and unintentional injuries and psychiatric and behavioral disorders. Out of 2267 DALYs per 100000 people, 780 (34.5%) years are years lived with disability (YLD). This fact highlights the role of secondary prevention alongside primary prevention for cardiovascular diseases (2).

Cardiac rehabilitation (CR) includes all the activities aimed at helping patients with cardiovascular disease to achieve their optimal physical, mental and social conditions, so that they may pursue their normal roles in the society and maintain an active life. Thus, the objective of CR is to reduce recurrence of disease and its mortality, improve clinical improvement through controlled exercises, modify lifestyle and educate about health. Exercise, lifestyle changes such as quitting smoking, blood lipid control, blood pressure control, weight control and treating diabetes, as well as psychological instructions are among interventions which efficiently influence patient’s relative recovery. Studies indicate that as a result of these interventions, CR reduces recurrence and disability, and improves quality of life and reduces mortality considerably as well (3-6).

Despite the positive outcomes reported with CR, it has been different from one region or country to another, and even different hospitals of one country have observed different results. These differences may be due to the content of CR program. On the other hand, CR programs with identical content may differ in terms of frequency and duration, and these differences will undoubtedly account for the observed discrepancies in terms of effect on cholesterol, High-density lipoprotein (HDL), Low-density lipoprotein (LDL) and blood pressure (3-12).

## 2. Objectives

Since no study has been conducted so far to evaluate the rehabilitation programs conducted at this center with their impacts on different health factors, as well as the fact that the programs are conducted relatively differently and the majority of patients are post-operative cardiac patients, we designed and conducted the present study.

## 3. Patients and Methods

The Imam Ali CR Center is located in Kermanshah in western Iran. It has initiated its activity since 2001 and is currently the exclusive CR center in western Iran, covering patients even from neighboring provinces. This is a retrospective cohort study on the data from Imam Ali Cardiac Rehabilitation Center. The participants included all patients who were referred to this CR center from 2001 to 2008 and completed its programs.

The CR program consists of medical and pharmaceutical evaluation, supervised exercise, and education and consultation for cardiovascular risk factors modification. The educational sessions address nutrition, medical, exercise (mostly endurance including threadmil, walking and stationary bicycle) and psychological issues. The program is presented as a comprehensive course (three sessions per week for two months, with a total of 26 sessions) or a short course (one session per week for two months, with a total of 10 sessions).

At the beginning of each course, the patients are evaluated for serum lipids, body mass index, waist circumference, blood pressure, depression, anxiety, smoking, diabetes, and exercise capacity in metabolic equivalents (METs) through maximal symptom limit exercise test. For the calculation of METs, we used modified Bruce protocol. Their information was recorded in their medical files. Once the CR program was completed, these evaluations were repeated and the changes were recorded again. All laboratory tests for blood lipids were performed in the laboratory of Imam Ali Hospital. In this study, data were extracted from patients’ medical files. Moreover, cases of readmission for cardiac problem and mortality (all-cause mortality) were identified through telephone calls made to patients or their families. All patients who had been readmitted were invited to refer to Imam Ali CR Center with their medical records. If the patients had moved from their previous address, we tried to locate them via friends or the telephone directory. Finally, those patients who could not be traced were searched for in the death registry of Kermanshah Province so that if expired, their date of death was recorded.

In the present study, we classified patients as hypertensive when they declared being hypertensive and received treatment for hypertension, or those with diastolic and systolic blood pressures (at the beginning of the program) of equal to or more than 90 and 140 mmHg, respectively. Regarding diabetes, patients were labeled diabetic if they declared to be diabetic and received insulin or oral medicine, or those with fasting blood sugar levels of equal to or more than 126 mg/dL at the beginning of the program.

We used Beck’s scale for diagnosis of depression. The patients were categorized in four groups in terms of severity of depression: no depression (0-9), mild depression (10-15), moderate depression (16-23), and severe depression (24-63). Spielberg’s scale was used for anxiety, categorizing patients as having no stress or mild stress (20-32), moderate stress (33-52), or severe stress (53-80). While Spielberg’s questionnaire can evaluate both the state and the trait of anxiety, in order to evaluate the effect of 2-3 months of the CR program we only used the “state” part.

In the present study, changes in anthropometric characteristics and cardiovascular risk factors, which constituted a continuous quantitative measurement, were assessed using paired t-test on STATA software. The relationship between variables and readmission (due to cardiac diseases) was evaluated using logistic regression for nominal or ranked variables. For the continuous quantitative variables, the mean values in two groups (with and without readmission) were compared using t-test (unequal variance). In order to evaluate mortality, first the survival rate curve was plotted for all patients. Considering the small number of expired patients during the study period, it was impossible to study the relationship between different variables and patient mortality using a multivariate model and therefore we used univariate cox proportional hazard method to determine the relationship between major variables and death. P values < 0.05 were considered significant for all analyses.

The present study was approved by ethics committee of Kermanshah University of Medical Sciences in 2009. After complete follow-up of patients till next event (hospital admission and/or death) we deleted all identifiers such as name, family name and father’s name.

## 4. Results

A total of 581 patients were referred to Imam Ali Center for CR from 2001 to 2008. Out of these patients, 504 completed the program; however, five patients were excluded from the study because of missing demographic data. Thus, a total of 499 patients were analyzed, consisting of 383 men and 116 women.

The men and women were not significantly different in terms of mean age; however, the number of non-smokers, the illiterate, and patients with hypertension and diabetes were significantly higher among women (Table 1). The majority of patients (87.8%) had been referred to the rehabilitation center following coronary artery bypass grafting surgery (CABG) or percutaneous coronary intervention (PCI); these patients constituted 66.4% of women, which was significantly lower compared to men (Table 1).

**Table 1.** Comparison of Demographic and Risk Factors Among Men and Women Participating in the Cardiac Rehabilitation Program^a,b^

Demographic and Risk Factors	Male (n = 383)	Female (n = 116)	P value	Total (n = 499)
**Age**	53.9 ± 9.0	54.8 ± 8.0	0.32	54.1 ± 8.8
**Smoking**			< 0.001	
Never	26.1	89.4		41.1
Cessation	68.4	8.9		54.3
Active	5.5	1.8		4.6
**Married**	98.4	80.8	< 0.001	94.3
**Education**			< 0.001	
Illiterate	9.9	38.8		16.6
Elementary	34.7	40.5		36.1
Diploma	25.3	12.9		22.4
University	30.1	7.8		24.9
**Indication**			< 0.001	
CABG/PCI	94.3	66.4		87.8
CAD	5.0	24.1		9.4
Valve surgery	0.7	5.2		1.8
High risk for CAD	0.0	4.3		1.0
**Diabetes**	20.4	35.3	0.001	23.9
**Hypertension**	35.0	54.2	< 0.001	48.5
**Cardiac Rehabilitation Type**			0.004	
10 sessions	13.8	4.3		11.6
26 sessions	86.2	95.7		88.4

^a^ Abbreviations: CABG, coronary artery bypass graft; CAD, coronary artery diseases, PCI, percotaneous coronary intervention.

^b^ Data are presented as mean ± SD or %.

### 4.1. Improvement in Risk Factors and Exercise Capacity

A comparison of risk factors and exercise capacity before and after rehabilitation indicated that all anthropometric measurements, systolic and diastolic blood pressure, blood lipids (except HDL), depression, anxiety, and exercise capacity improved after rehabilitation program (P < 0.05 for all cases) (Table 2). This improvement except for HDL was observed for both sexes (data is not presented in tables). In addition, the type of CR (8 sessions or 24 sessions) did not affect the magnitude of improvement considerably (P > 0.05 for all cases).

**Table 2.** Comparison of Risk Factors and Exercise Capacity Before and After Cardiac Rehabilitation^a,b^

Variables	Before Rehabilitation	After Rehabilitation	P value
**Weight, kg**	73.5 ± 10.3	71.8 ± 10.0	< 0.001
**Body Mass Index, kg/m** ^**2**^	26.5 ± 3.8	25.9 ± 3.6	< 0.001
**Waist, cm**	95.4 ± 9.3	92.1 ± 9.0	< 0.001
**Systolic Blood Pressure, mmHg**	128.6 ± 17.7	117.3 ± 11.7	< 0.001
**Diastolic Blood Pressure, mmHg**	82.2 ± 10.6	76.2 ± 7.9	< 0.001
**Depression (Beck score) ** ^**c**^	14.0 ± 8.7	9.3 ± 6.5	< 0.001
**Anxiety (Spielberg score) ** ^**c**^	40.2 ± 11.0	36.7 ± 8.9	< 0.001
**Exercise Capacity (METs)**	9.4 ± 2.6	11.4±2.7	< 0.001
**Total Cholesterol, mg/dL**	211.3 ± 47.0	190.4 ± 42.9	< 0.001
**LDL Cholesterol, mg/dL**	132.2 ± 36.0	116.6 ± 37.7	< 0.001
**HDL Cholesterol, mg/dL**	41.2 ± 12.6	41.8 ± 13.5	0.31
**Triglyceride, mg/dL**	192.6 ± 81.9	161.4 ± 73.2	< 0.001

^a^ Abbreviations: HDL, high-density lipoprotein; LDL,low-density lipoprotein, METs, metabolic equivalents.

^b^ Data are presented as mean ± SD.

^c^ Data pertaining to depression and stress before and after program existed for only 431 and 366 patients, respectively.

### 4.2. Readmission and Its Related Factors

Out of 499 patients referred for rehabilitation, 40 patients were readmitted to hospital for cardiac reasons; one patient was readmitted prior to initiation of rehab program, and was therefore excluded from the analysis. In the present study, factors of hypertension, smoking, education level, age, depression, stress, and marital status were not related to readmission (Table 3). On the other hand, female sex (OR = 2.40; 95%CI: 1.22-4.68) and history of diabetes (OR = 2.04; 95%CI: 1.04-4.02) significantly increased the risk of readmission (Table 3). Furthermore, a comparison of mean anthropometric measurements, exercise capacity, and mean values of blood lipids in patients with or without readmission indicated that readmitted patients had larger anthropometric measurements at the beginning and the end of CR. Although the exercise capacity of readmitted patients was initially lower compared to those who were not readmitted, no statistically significance difference in exercise capacity was observed at the end of the rehabilitation program (Table 4).

**Table 3.** Relationship Between Different Variables and Readmission After Completion of CR^a^

Variables	OR	CI 95%
**Age ≥ 55**	0.65	0.33-1.26
**Gender (Female)**	2.4	1.22-4.68
**Education**		
illiterate	1.00	-
elementary	0.59	0.24-1.45
Diploma	0.47	0.16-1.36
University	0.96	0.39-2.37
**Marriage**	0.47	0.17-1.30
**Diabetes**	2.04	1.04-4.02
**Hypertension**	0.89	0.43-1.80
**Cardiac Rehabilitation Type**		
10 sessions	1.00	-
26 sessions	2.19	0.51-9.47
**Smoker (at start of program)**		
Never	1.00	-
Cessation	0.62	0.31-1.21
active	0.88	0.19-4.05
**Indication**		
CABG/PCI	1.00	-
CAD	2.70	1.16-6.26
Valve surgery	-	
High risk for CAD	3.28	0.36-30.27
**Depression status**		
Without depression	1.00	-
Mild depression	0.90	0.38-2.10
Moderate depression	1.30	0.52-3.20
Severe depression	1.63	0.58-4.60
**Anxiety status**		
Without or mild anxiety	1.00	-
Moderate anxiety	0.80	0.32-2.00
Severe anxiety	1.06	0.29-3.84

^a^ Abbreviations: CABG, coronary artery bypass graft; CAD, coronary artery diseases; CI, confidence interval; CR, cardiac rehabilitation; OR, odds ratio; PCI, percutaneous coronary intervention.

**Table 4.** Comparison of Anthropometric Measurements and Exercise Capacity in Patients With and Without Readmission ^a,b^

Variables	With Readmission	Without Readmission	*P* value
**Weight before CR, kg**	76.6 ± 10.3	73.2 ± 10.3	0.04
**Weight after CR, kg**	74.9 ± 10.0	71.6 ± 9.9	0.05
**BMI before CR, kg/m** ^**2**^	28.3 ± 4.0	26.3 ± 3.7	0.004
**BMI after CR, kg/m** ^**2**^	27.6 ± 4.0	25.8 ± 3.6	0.007
**Waist before CR, cm**	100.0 ± 9.2	95.0 ± 9.3	0.002
**Waist after CR, cm**	96.0 ± 8.6	91.7 ± 9.0	0.004
**Exercise capacity before CR (METs)**	8.6 ± 2.8	9.6 ± 2.6	0.04
**Exercise capacity after CR (METs)**	10.9 ± 2.9	11.4±2.7	0.33

^a^ Abbreviations: CR, cardiac rehabilitation; METs, metabolic equivalents.

^b^ Data are presented as mean ± SD.

### 4.3. Survival Rate in Referred Patients

After a maximal follow-up period of 6.3 years (median = 2.99 years, mean = 3.27 ± 1.74 years), only eight patients expired (survival rate = 97.5 years; 95% CI = 94.7-98.8), consisting of seven men and one woman. Among these, six patients were referred to the CR center following CABG/PCA. None of the variables in our study were significantly related to survival rate in the univariate analysis.

## 5. Discussion

The present study indicated that CR in combination with other interventions can efficiently improve the modifiable cardiovascular risk factors, except HDL. Furthermore, it was indicated that history of diabetes and female sex are two risk factors for readmission in patients referred for CR.

Previous studies have indicated different results for the effect of comprehensive CR on mean blood lipids values. While some clinical trials have suggested that rehabilitation reduces total cholesterol effectively (7-10), others have failed to indicate a statistically significant effect (11, 12). This is also true for LDL and triglyceride levels (10-12). Most previous studies have indicated that CR does not affect HDL levels significantly (7, 12, 13). Similarly, the present study found that CR reduces all blood lipids except HDL, and this is a mechanism for reducing the risk of cardiovascular disease and death in these patients. Nevertheless, it must be noted that despite all these reports, which sometimes differ regarding the effect of CR on blood lipids, meta-analysis by Taylor et al. (2004) indicated that rehabilitation improves total cholesterol, LDL and triglyceride levels while leaving HDL nearly unchanged (5). In fact exercise and possible changes in nutritional pattern of patients during rehabilitation are among major factors which reduce blood lipids.

Several points must be made about the anthropometric changes. In the present study, all anthropometric measurements (weight, waist circumference and body mass index) improved. Although relatively small (about 2%), the changes were statistically significant. As a matter of fact, many CR programs integrate weight control and thus it is expected that patients lose 10% weight (1-2 pounds per week) over a six-month period (14). However, it is noteworthy that many reports indicate that this weight loss is not feasible in short term (15, 16) and even in some cases, particularly for obese and overweight patients, the patients may initially gain some weight (17, 18). Previous studies have reported a maximum of 1% to5% weight loss (19-21) which is consistent with our findings.

Our study showed that CR has an impressive blood pressure–lowering effect: 11.3 mmHg for systolic and 6.0 mmHg for diastolic blood pressure. This study confirms the findings of previous studies that CR reduces both systolic and diastolic blood pressure (9, 22). A meta-analysis including 54 clinical trials showed that after aerobic exercise program, systolic and diastolic blood pressure reduced significantly (3.9 and 2.6 mm Hg respectively). However, our findings are in contrast to another meta-analysis (5) that showed exercise based cardiac rehabilitation doesn't improve diastolic blood pressure. The reasons for this discrepancy can be attributed to methodological differences among different studies. Findings of one study indicate that while exercise alone is effective in reducing blood pressure, the addition of a behavioral weight loss program significantly augments the efficacy of aerobic training (23). Another study showed that diastolic blood pressure changes positively correlate with serum cholesterol and insulin resistance (24) which can be enhanced during comprehensive CR programs. The effect of CR on blood pressure needs to be further investigated in future prospective studies including proper control groups.

An important finding of the present study is the improved exercise capacity by 25%. After cardiac events, the METs level has been a shown to be a significant predictor of final prognosis of patients (25). According to the American Heart Association report, CR for 3 to 6 months generally increases exercise capacity by 11% to 36% (26). Improvement of exercise capacity after CR has been shown in numerous studies (13, 27-30).

The present study shows an improvement in depression and anxiety following CR program which is in line with findings of previous studies (21, 31, 32). Enhancement of anxiety and depression have a major impact on medical outcomes in patients with cardiovascular disease by reducing morbidity and mortality as well as improving the quality of life (33). It is important to note that we evaluated the state of anxiety in a way that we evaluates how the patients feel at the moment of evaluation. The responses can vary with the feelings of patients. In fact there are different reasons for observed improvement in depression and anxiety of patients. One important reason can be related to the content of program. We run the CR program within a group of patients and therefore it provides a positive atmosphere for better interaction with each other. In addition, most of people referred to this center are after CABG. Such patients have passed the recovery period and CR program can improve their feeling of well-being.

We also studied the factors related to readmission of patients after completion of CR program. Numerous studies have dealt with the effect of CR on readmission (34-36). It seems that factors influencing readmission in patients undergoing CR cannot be very different from factors influencing readmission after a cardiovascular event. Most studies have incriminated diabetes as a factor affecting the survival rate and subsequent hospital admissions of different types of cardiac patients (37, 38). Similarly, some studies have reported poorer prognosis and higher risk of readmission for female patients (38, 39). This is quite consistent with our findings which indicate a higher rate of readmission for female and diabetic patients.

The survival rate was 97.5% in the present study. As a matter of fact, although previous studies have yielded controversial results regarding the effect of CR on survival rate in patients with cardiac events, most recent studies suggest a positive role for CR in terms of reducing mortality (4, 5, 40, 41). Since our study does not entail a control group, we were unable to compare the survival rate in our patients with those who did not take CR, yet comparing our findings with other studies suggests that survival in our patients (mostly patients who underwent CABG/PCI) is relatively higher compared to patients who do not participate in cardiac rehabilitation (42, 43).

### 5.1. Strengths and Weaknesses

The present study is the largest study conducted in western Iran, where the only CR center covers Kermanshah and its neighboring provinces, thus its findings are of immense value. Nonetheless, due to the short duration of follow-up and the low incidence of outcomes, it cannot identify the influential factors, which require further studies with longer durations of follow-up. On the other hand, we were unable to control the treatments that patients received during rehabilitation (which were often changed by physicians) and include them in our analysis. We are not able to determine the exact contribution of changes in medication in the observed improvements. In addition, because of no control group one may discuss that the observed improvement in risk factors might be the effect of factors other rather than CR. Prospective studies with inclusion of control group may circumvent this problem to a large extent. In addition, due to the small number of cases for some variables (e.g. active smokers among women), some of the statistical tests did not have sufficient power.

In spite of the low popularity of CR programs among cardiologists in countries like Iran, such programs can efficiently reduce cardiovascular risk factors and improve exercise capacity in patients with heart disease. The outcomes of these programs in Imam Ali CR center in Kermanshah are comparable to CR programs in other centers. Such findings are in line with other studies to show that cardiologists’ fears are not rational. Though not certainly, we can believe that CR plays an important role in reducing the risk factors of CVD. History of diabetes as well as being female both increased the risk of readmission for cardiac reasons.
